# Induction of apoptosis with tobacco smoke and related products in A549 lung epithelial cells *in vitro*

**DOI:** 10.1186/1476-9255-3-3

**Published:** 2006-03-21

**Authors:** Lindsay Ramage, Amanda C Jones, Clifford J Whelan

**Affiliations:** 1 School of Life Sciences, University of Hertfordshire, College Lane, Hatfield, Herts., AL10 9AB, UK

## Abstract

**Background:**

This study has investigated the ability of tobacco smoke, and ingredients of tobacco smoke, to induce apoptosis in the airway epithelial cell line A549.

**Method:**

A549 cells were treated with 80 μg/ml Tobacco smoke condensate (TSC), 10 mM Nicotine, 10 μM paraldehyde, 10 μM hydrogen peroxide, 1 μM Taxol^® ^(Paclitaxel), 100%, 50% and 25% cigarette smoke extract (CSE). Following 4–48 h incubation apoptosis was measured morphologically following staining of cells with DAPI. TUNEL staining was also used to assess DNA damage after 24 and 48 h incubation. In addition, loss of mitochondrial cytochrome C and activation of Bax-α, early events in the apoptotic process, were measured after 4 h of incubation.

**Results:**

Incubation of A549 cells with vehicle, Taxol, TSC, nicotine, paraldehyde, hydrogen peroxide and CSE caused a time-dependent detachment of the cells from the flask between 6 and 48 h. DAPI staining revealed that the cells remaining adhered to the flask appeared healthy whereas some of those that had detached appeared to be either apoptotic or indeterminate. Treatment with Taxol, TSC, nicotine, paraldehyde, hydrogen peroxide and CSE caused a significant increase in the number of apoptotic cells. Similarly, treatment with Taxol, TSC, nicotine, hydrogen peroxide and CSE caused a significant increase in the number of apoptotic cells among the cells that had detached from the culture plate. After 4 h of incubation, Taxol, TSC, hydrogen peroxide and CSE caused a significant reduction in mitochondrial cytochrome C and an increase in cytosolic cytochrome C. At the same time point, hydrogen peroxide and CSE significantly increased the concentration of Bax-α in the mitochondria.

**Conclusion:**

Tobacco smoke initiates apoptosis in A549 airway epithelial cells as a result of mitochondrial damage and that this results in a cell detachment and full apoptosis. This effect appears to result from factors in tobacco smoke other than nicotine and may result from free radical activity. However, additional stable factors may also be involved since the free radical content of TSC is likely to be low.

## Introduction

Cigarette smoking has been associated with inflammatory diseases of the lung, particularly chronic obstructive pulmonary disease (COPD). These changes are characterised by increased vascular permeability, neutrophil sequestration, and increased cytokine, oxidant and protease production in the [1,2] lung. Tobacco smoke is a mixture of more than 4000 components including carcinogens, oxidants and aldehydes, all of which have the potential to cause inflammation and damage cells.

Oxidants are thought to play a major role in cell injury induced by tobacco smoke since each puff of tobacco smoke contains approximately 10^17 ^oxidant molecules [3]. Although many of these are short lived the condensate formed in the airspaces continues to produce oxidants after smoking cessation [4]. Aldehydes have been implicated in the toxic effects of cigarette smoke, in particular acrolein. It is thought that aldehydes mediate their effects by increasing the intracellular oxidative activity of the cells [5]. However, the nature and mechanism of tobacco smoke-induced cell damage and death is not clear.

Cigarette smoke has been associated with damage to the alveolar epithelium. In particular it can induce suppression of cell proliferation, increased detachment of cells, DNA strand breaks, and reduced surfactant production [5-8]. Breaking of DNA strands has been associated with apoptosis of alveolar cells. There is conflicting evidence for the induction of apoptosis in airway epithelial cell lines following tobacco smoke exposure although where apoptosis is observed antioxidants ameliorate any apoptosis seen, a finding consistent with the hypothesis that free radicals in smoke are responsible for any apoptosis observed [3]. One study suggested that these effects are reversible [7] but it has also been shown that exposure of airway epithelial cells to cigarette smoke did not cause apoptosis but induced cell death by necrosis only [9].

The aim of the present study was to investigate the effect of tobacco smoke and some of its components on apoptosis in A549 cells, an epithelial cell line. Apoptosis was assessed by methods that measure both early and late events in the apoptotic process in order to determine the likely pathway by which apoptosis, induced by tobacco smoke, occurs and to determine whether early damage results in cell death. To this end, apoptosis was determined by cell morphology following DAPI staining and by TUNEL labelleing of fragmented DNA, both relatively late events. In addition, cytochrome C release from mitochondria and activation of the proapoptotic factor Bax was measured, both early events in the process of apoptosis, 4 h after exposure of cells to the treatments used.

## Materials and methods

All materials were obtained from Sigma-Aldrich (Poole, UK) unless otherwise stated.

### Cell culture

A549 human lung epithelial cells were obtained from the European cell culture collection (ECACC). The cells were maintained in Nutrient mixture F12 Ham supplemented with 10% fetal bovine serum, 2 mM L-glutamine (Life Technologies, Paisley, UK), 50 μg/ml penicillin (Life Technologies), 50 μg/ml, streptomycin (Life Technologies) and 2.5 μg/ml amphotericin B. Cells were cultured at 37°C in a humidified atmosphere containing 5% CO_2_. Cells were grown in tissue culture flasks until they were 80% confluent before trypsinisation with 1× trypsin-EDTA (Life Technologies, Paisley, UK) and splitting.

### Cell treatments

Cells were treated for 4, 24, or 48 hours. Cell treatments were directly prepared into media (unless a specified vehicle was also used) to give the final concentration stated. These were, 80 μg/ml Tobacco smoke condensate (TSC; contains 1% DMSO in final volume), 10 mM Nicotine, 10 μM paraldehyde (also known as acetaldehyde trimer), 10 μM hydrogen peroxide, 1 μM Taxol^® ^(Paclitaxel), 100%, 50% and 25% cigarette smoke extract (CSE). Untreated cells in serum free media were used as controls and those exposed to 1% DMSO as a vehicle control. All solutions were used within 30 minutes of preparation. TSC was obtained from Dr Eian Massey, (British American Tobacco, Southampton, UK) and was prepared from cigarettes which were smoked on 20 port Borgwaldt rotary smoking engine under ISO smoking conditions (a 35 ml puff, over a 2 second duration taken once per minute). The smoke particulate matter was trapped on a Cambridge filter pad, eluted with DMSO within 30 minutes of production, adjusted to a concentration of 24 mg/ml total particulate matter (containing 2.05 mg/ml nicotine) and frozen at -70°C until use. CSE was prepared from the smoke of 1 cigarette bubbled through 50 ml serum free media using a vacuum pump and filtered (0.22 μM filter). 100% CSE contains 0.051 (+/-0.01) mg/ml nicotine (determined by HPLC). The concentrations of TSC and CSC used in the present study were chosen because they had no cytotoxic effect as determined by the MTT assay (data not shown) and because they represented concentrations likely to be experienced *in vivo *(based on a blood level of 10^-8 ^M nicotine).

### Detachment of cells from culture flask

During exposure to the various treatments the number of cells detaching was measured in the cell supernatant. Cells were grown in 24 well plates at a seeding density of 10^5 ^cells/ml in complete media overnight; cells were washed in PBS and treated for up to 48 hours in serum free media. An aliquot of the culture medium (0.5 ml) was removed and treated with trypan blue. The total number of detached cells was determined by counting in a haemocytometer and cell viability of detached cells was measured by trypan blue exclusion. Viability of the detached cells was >95% in all cell cultures after treatment.

### DAPI staining

Cells were grown in 24 well plates at a seeding density of 10^5 ^cells/ml of complete media overnight. Cells were washed in PBS and treated for 24 or 48 hours in serum free media. Samples of detached cells were deposited on microscope slides by centrifugation at 750 rpm for 10 minutes (Cytospin 2, Thermo Shandon). Cells were fixed with 3.7% formaldehyde prior to washing with PBS. Washed cells were then stained with 1 μg/ml DAPI for 15 minutes. Slides were viewed with a fluorescent microscope at 340–380 nm and ×1000 magnification (Nikon ERFD-3 microscope attached to Nikon FDX35 camera). Three hundred cells were counted per slide randomly and determined to be normal or apoptotic depending on morphological characteristics. A cell was considered to be normal if it had typical morphology with smooth nuclear and cellular membranes. Apoptotic cells had condensed nuclear chromatin with or without apoptotic bodies. "Indeterminate" cells had disrupted nuclear and/or cellular membranes, or "cauliflower" shaped nuclei (particularly associated with Taxol treatment; see additional file). Thus, the percentage of normal, apoptotic or indeterminate detached cells was obtained. These data were converted to absolute cell numbers to correct for any treatment-related detachment of cells from the culture.

### Terminal deoxynucleotidyltransferase-mediated dUTP nick end labelling assay (TUNEL)

Cells were grown in 24 well plates at a seeding density of 10^5 ^cells/ml in complete media overnight. Cells were washed in PBS and treated for 24 or 48 hours in serum free media. The number of detached cells in the culture medium was determined and a sample deposited onto microscope slides by centrifugation at 750 rpm for 10 minutes (Cytospin 2, Thermo Shandon). Cells were fixed in 4% methanol-free formaldehyde (Polysciences, UK) for 10 minutes. Permeablisation of the cells was carried out by incubation of the fixed cells with 0.2% Triton X. Positive control cells were then created by treatment with DNAse I (Promega, UK) for 10 minutes, to break up the DNA. Slides were washed in PBS between each of these steps. Using the DeadEnd fluorimetric TUNEL system (Promega) slides were incubated with equilibrium buffer prior to addition of nucleotide mix containing equilibration buffer, labelled nucleotide mix and rTdT enzyme for 60 minutes at 37°C. Cells were washed in SCC buffer and stained with DAPI. For each treatment 300 cells stained with DAPI (340–380 nm) were randomly counted and assessed for FITC (450–490 nm) staining. The number of FITC positive cells was expressed as a percentage of the total number counted and used to determine the total number of TUNEL positive cells in the detached cell population.

### Cytochrome C detection

Cell samples were prepared using the method described in the Cytochrome C release kit (Oncogene). Cells were grown in 75 cm^2 ^flasks at a seeding density of 5 × 10 ^7 ^cells in complete media overnight. Cells were washed in PBS and treated for 4 hours in 20 ml serum free media. Both adherent and detached cells were pooled prior to cytochrome C extraction, since little detachment occurred in the first 6 hours of incubation. Culture supernatants were removed and centrifuged at 600 × g for 5 minutes at 4°C to recover detached cells. Adherent cells were washed with PBS and detached using trypsin EDTA. The detached cells were added to the adherent cells and centrifuged. The resulting pellet was washed in ice cold PBS. Pelleted cells were re-suspended in cytosolic extraction buffer (Cytochrome C release kit, Oncogene) and incubated on ice for 10 minutes. The cells were then homogenised and the resulting suspension was transferred to a 1.5 ml eppendorf tube and centrifuged at 700 × g for 10 minutes at 4°C. The supernatant was removed and centrifuged at 10,000 × g for 30 minutes at 4°C to recover the mitochondria. The resulting supernatant was removed and kept as the cytosolic fraction and the pellet was re-suspended in mitochondrial buffer. Protein assays were performed to equalise samples prior to use by the detection assays

Cytochrome C Western blots were performed on the cell fractions for hydrogen peroxide treated cells and untreated controls. 10 mg aliquots of mitochondrial and cytosolic protein were separated on 10% SDS-Page gels, transferred to nitrocellulose and detected using specific antibody provided in cytochrome C release kit.

Cytochrome C was determined by ELISA (R and D Systems, Abingdon, UK) on mitochondrial and cytosolic fractions of cells (protein concentration 200 μg/ml). Cytochrome C was detected with in the range 0.62–20 ng/ml and minimum level of detection for this kit is 0.30 ng/ml. Cytochrome C was expressed as ng/mg protein.

### Bax-α Assay

Cell samples were prepared using the method described in the cell cytosol/mitochondria fractionation kit (Calbiochem). Cells were grown in 75 cm^2 ^flasks at a seeding density of 5 × 10^5 ^cells in complete media overnight, cells were washed in PBS and treated for 4 hours in 20 ml serum free media. Both adherent and detached cells were used in this assay since little detachment occurred in the first 6 hours. Supernatants were removed and centrifuged at 600 × g for 5 minutes at 4°C. Adherent cells were washed with PBS and detached using trypsin EDTA. The detached cells were added to the supernatant cells and centrifuged. The resulting pellet was washed in ice cold PBS. Pelleted cells were re-suspended in cytosolic extraction buffer (cytosol/mitochondria fractionation kit) and incubated on ice for 10 minutes. The cells were then homogenised and the resulting suspension was transferred to 1.5 ml eppendorf tube and centrifuged at 700 × g for 10 minutes at 4°C. The supernatant was removed and centrifuged at 10,000 × g for 30 minutes at 4°C. The resulting supernatant was removed and kept as the cytosolic fraction and the pellet was re-suspended in mitochondrial buffer. Protein assays were performed and the protein content of all samples adjusted to 200 μg·ml^-1 ^prior to use by detection assays. Bax-α was measured in mitochondrial and cytosolic extracts using? a Bax-α ELISA kit (R and D Systems, Abingdon, UK). Bax-α was detected with in the range 0.62–20 ng/ml.

### Statistical analysis

All experiments were repeated three times each on different days and as appropriate the mean and s.e. mean were calculated. For the case of cell counts after DAPI and TUNEL staining the mean numbers per slide were counted as n = 1. Statistical significance was carried out using one way ANOVA and Dunnetts post hoc test. Differences were considered significant when p < 0.05.

## Results

### Morphological changes after tobacco smoke exposure

**Figure 1:**
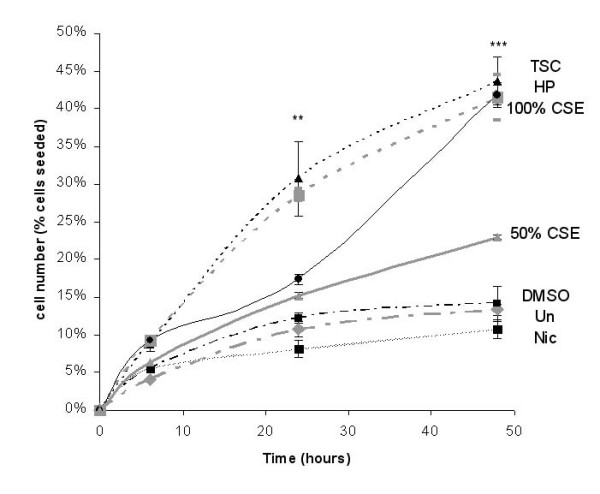
Detachment of A549 cells from the culture vessel counted over 48 hours. Cells were seeded at a density of 10^5 ^per ml. Cells were untreated (control), or treated with TSC (80 μg/ml), nicotine (Nic; 1 mM), hydrogen peroxide (HP; 10 μM), and 100% and 50% CSE. Asterisks denote statistical significance from control cells, ***, p < 0.001, **, p < 0.01. Each point is the mean ± s.e. mean of data from at least 3 separate experiments.

Changes in the morphology of A549 cells were analysed at times from 0 – 48 h after incubation with Taxol (1 μM; positive control for apoptosis), TSC (80 μg/ml), nicotine (1 mM), paraldehyde (10 μM), hydrogen peroxide (10 μM), CSE (50 and100%), or vehicle. It was noted that many cells detached from the flask over time and there was a time-dependent detachment of the cells after 6 hours (Figure 1). CSE also caused a concentration-dependent detachment of A549 cells with 100% CSE causing approximately 2-fold increase in detachment, compared to 50% CSE, after 48 hours (Figure 1).

**Figure 2:**
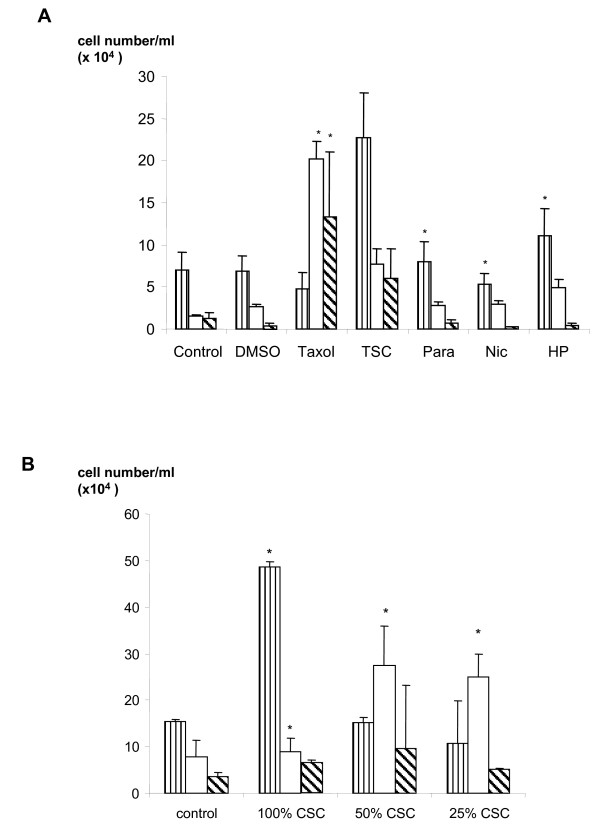
Graph of cell distribution in detached A549 cells after 48 hours of treatment measured by morphological analysis by DAPI staining. Data shown the number of normal, apoptotic or indeterminate cells among the detached cell population as measured by morphological assessment. Cells were seeded at a density of 10^5 ^cells per well, prior to treatment in serum free media. (A) Cells were untreated (control) or treated with DMSO (1%), Taxol (1 μM), TSC (80 μg/ml), paraldehyde (Para; 10 μM), nicotine (Nic; 1 mM) and hydrogen peroxide (HP; 10 μM). (B) Cells were untreated (control) or treated with 100%, 50% and 25% CSE. Vertically striped bars show "normal", open bars show "apoptotic" and hatched bars show "indeterminate" cells/ml of the culture and are the mean ± s.e. mean of at least 3 determinations. Asterisks denote statistical significance (p < 0.05) from control cells showing the same morphology.

When cells were stained with DAPI and observed under the microscope little apoptosis was seen in the cells that remained adherent to the plate (data not shown). However, marked morphological changes were seen in the detached cells. When the slides were viewed it became apparent that there was not a "clear cut" observational change between normal and apoptotic nuclei. Some cells had nuclei that were condensed or fragmented and these were termed apoptotic, while others appeared to have normal morphology. A third group of cells appeared to be neither normal, nor apoptotic were termed "indeterminate". Furthermore, in the case of Taxol treated cells, many had a 'cauliflower' shape [see additional file 1]. After 48 hours Taxol (1 μM), paraldehyde (10 μM), nicotine (1 mM), hydrogen peroxide (10 μM) and CSE (25–100%) caused a significant (p < 0.05) increase in the number of apoptotic cells (Figures 2a and 2b). While treatment with Taxol also caused a significant (p < 0.05) increase in the number of "indeterminate" cells. Interestingly, there appeared to be a discrepancy between cells treated with 100% and 50% CSE in that the higher concentration apparently had no effect on the cell morphology while the lower concentration has a much greater effect on cell morphology and apoptosis.

### DNA damage induced after tobacco smoke exposure

**Figure 3:**
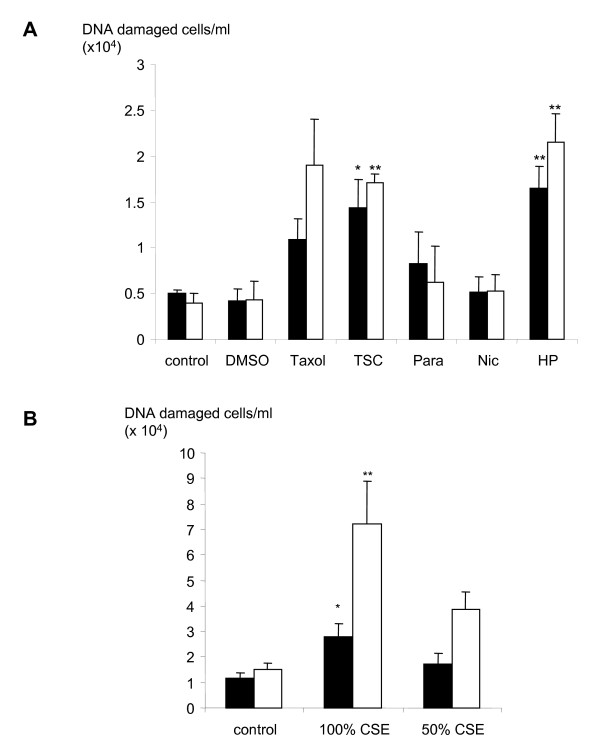
The number of DNA damaged cells in the detached cell population after 24 or 48 hours of treatment as measured by TUNEL staining. Values shown are the absolute number of TUNEL positive cells in the detached cell population. Cells were seeded at a density of 10^5 ^cells per well, prior to treatment in serum free media. (A) Cells were untreated (control) or treated with DMSO (1%), Taxol (1 μM), TSC (80 μg/ml), paraldehyde (Para; 10 μM), nicotine (Nic; 1 mM) and hydrogen peroxide (HP; 10 μM). (B) Cells were untreated (control) or treated with 100%, 50% and 25% CSE. Closed bars show cells treated for 24 hours and open show cells treated for 48 hours. Bars are the mean ± s.e. mean of data of TUNEL positive cells/ml of culture from at least 3 separate experiments. Asterisks denote statistical significance from control cells of same time point, *, p < 0.05 and **, p < 0.01.

TUNEL staining was used to examine DNA damage in A549 cells 24 and 48 hours after treatment (Figures 3a and 3b). Since the number of detached cells varied depending on the treatment used, TUNEL positive cells are expressed as the number of cells per ml of culture medium. The data obtained show that the number of cells with DNA damage was significantly increased following treatment with TSC (80 μg/ml; p < 0.05), hydrogen peroxide (10 μM; p < 0.01), Taxol (1 μM; p < 0.01) and 100% CSE (p < 0.05) at 24 hours. The number of TUNEL positive cells was further increased after 48 hours incubation. There was also a concentration-dependent increase in cells staining TUNEL positive after treatment with 100% CSE over that seen following treatment with 50% CSE. Thus, CSE appeared to cause a concentration-related increase in TUNEL positive cells.

### Cytosolic translocation of cytochrome C after tobacco smoke exposure

**Figure 4:**
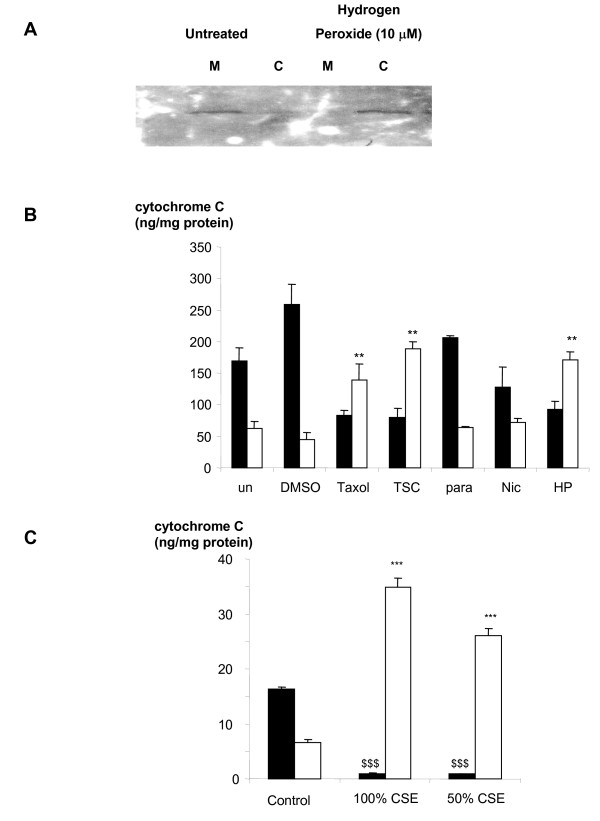
Cytochrome C levels within cytosolic and mitochondrial fractions of cells after 4 hours of treatment. (A) Western blotting of cytochrome C. Cells were treated with hydrogen peroxide (10 μM) and untreated control. (B) Lysates were measured by ELISA. Cells were untreated (control) or treated with DMSO (1%), Taxol (1 μM), tobacco smoke condensate (80 μg/ml), paraldehyde (10 μM), nicotine (1 mM) and hydrogen peroxide (10 μM). (C) Lysates were measured by ELISA. Cells were untreated (control) or treated with CSE (50 and 100%). Closed bars show mitochondrial fraction and open show cytoplasmic fraction. Bars are the mean ± s.e. mean cytochrome C/ml assay media from at least 3 separate experiments. Asterisks denote statistical significance from control cells of same cell fraction. **, p < 0.01 and ***; $$$, p < 0.001.

Cells were treated for 4 hours before cytosolic and mitochondrial fractions were prepared from the adherent and detached cells combined. Cytochrome C was measured by Western blotting and specific ELISA (Figures 4a, b and 4c). The concentration of cytosolic cytochrome C was increased following treatment with Taxol (1 μM; p < 0.01), TSC (80 μg/ml; p < 0.01) and hydrogen peroxide (10 μM; p < 0.01) after 4 hours (Figure 4b). In a separate experiment, (Figure 4c) CSE (50% and 100%) caused a significant (p < 0.001) loss of mitochondrial and increase in cytosolic cytochrome C after 4 hours of incubation.

### Increased concentrations of Bax-α after tobacco smoke exposure

**Figure 5:**
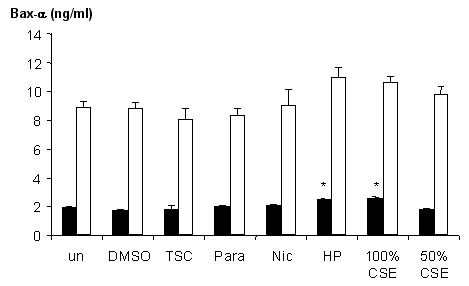
Bax-α levels within cytosolic and mitochondrial fractions of cells after 4 hours of treatment as measured by ELISA. Cells were untreated (control) or treated with DMSO (1%), tobacco smoke condensate (80 μg/ml), paraldehyde (10 μM), nicotine (1 mM), hydrogen peroxide (10 μM) and CSE (50 and 100%). Closed bars show mean Bax-α levels in the mitochondrial fraction and open show the cytoplasmic fraction. Bars are the mean ± s.e. mean Bax-a/ml assay medium from at least 3 separate experiments. Asterisks denote statistical significance from control cells of same cell fraction. *, p < 0.05.

In the present study, mitochondrial protein comprised approximately 20% of the total cellular protein. After 4 hours of incubation, Bax-α was detected in both the cytosolic and mitochondrial fractions. Following incubation with hydrogen peroxide, a significant increase in mitochondrial Bax-α (p < 0.05) was measured (from 2.06 ± 0.12 ng/ml assay medium to 2.62 ± 0.60 ng/ml assay medium) an increase of 27% (Figure 5). Similarly following treatment with CSE (100%) mitochondrial Bax-α was significantly increased (p < 0.05) by 33% from 2.06 ± 0.12 ng/ml assay medium to 2.75 ± 0.52 ng/ml assay medium (Figure 5). No other treatment caused a significant change in mitochondrial Bax-α. Similarly, none of the treatments used caused a significant change in cytosolic Bax-α (P > 0.05) although there was a trend for cytosolic Bax-α to be increased following treatment with hydrogen peroxide and CSE (100%)(Figure 5).

## Discussion

The aim of the present study was to investigate the effect of tobacco smoke and some of its components on apoptosis in A549 cells, an epithelial cell line. The concentrations of TSC, CSC and nicotine used in the present study were chosen because they represented concentrations likely to be experienced *in vivo *(based on a blood level of 10^-8 ^M nicotine [16]) and because they had no cytotoxic effect as determined by the MTT assay (data not shown). Similarly, the concentrations of hydrogen peroxide and paraldehyde were chosen in an attempt to replicate levels likely to be obtained following exposure to tobacco smoke. In an attempt to describe the mechanism of tobacco smoke-induced apoptosis, In addition to measuring end points of the apoptotic process (Nuclear condensation and DNA fragmentation) we also measured events that occur shortly after the initiation of apoptosis by oxidative stress (cytochrome C release and Bax activation). Initial experiments revealed that tobacco smoke condensate and fresh tobacco smoke caused a precipitate if serum was included in the media. Thus, we elected to omit the serum during the incubations. In addition, omission of serum will halt cell growth and division thus eliminating any inhibitory action of our treatments on cell growth. However, this practice did not adversely affect cell viability as measured by the MTT assay.

The data obtained in the present study show that tobacco smoke, but not nicotine, induced apoptosis as measured by cell morphology, and by the TUNEL method of assessing DNA damage, over a 48 period following exposure to these agents. Furthermore, tobacco smoke-induced apoptosis was accompanied by a time-dependent detachment of cells from the culture plate and the apoptotic cells were found in the detached cell fraction. The small degree of apoptosis seen also appeared to be preceded by a marked release of cytochrome C and activation of Bax from the mitochondria 4 h after treatment. However, because few cells detached from the culture over this period, cytochrome C and Bax measurements were made on all the cells in culture and further experiments are necessary to determine whether or not these changes occurred before or after detachment.

Apoptosis is characterised by morphological changes such as membrane "blebbing", nuclear condensation and fragmentation [10]. These morphological changes are a result of a cascade of biochemical changes occurring within the cell, resulting in activation of caspases and DNA fragmentation [11]. However, while it is possible to measure apoptosis by monitoring these early biochemical changes or end points such as DNA fragmentation, reports in the literature suggest that the choice of end point may have a marked effect on the magnitude of apoptosis measured. This difference may have relevance to the ability of tobacco smoke to induce apoptosis in airway epithelial cells since reports in the literature do not agree as to whether tobacco smoke causes apoptosis or necrosis. For example, one study measured the effects of tobacco smoke on A549 epithelial cells and concluded that tobacco smoke induced apoptosis when measured morphologically [5]. In contrast, a similar study also measured apoptosis morphologically and concluded that tobacco smoke induced necrosis rather than apoptosis in A549 cells [9]. Thus, in the present study, we elected to adopt a temporal approach to resolving this discrepancy by measuring both early and later markers of apoptosis.

Analysis of the cell morphology after exposure to tobacco smoke and the other treatments used, showed that many of the cells detached from the culture flask confirming the findings of others [6]. The observation, in the present study, that the majority of apoptotic cells had detached from the culture may explain the discrepancy between our data and that reported by others [9] since the latter study only measured apoptosis in the attached cells. Many of the detached cells could not easily be clearly categorised as normal or apoptotic and hence were labelled indeterminate. In particular, nuclei of Taxol treated cells were "cauliflower shaped". Since Taxol is an inhibitor of microtubule function [12], these changes may result from the cells becoming arrested in the early stages of mitosis (often prophase).

When apoptosis was measured by morphological assessment, we generally found that the percentage of apoptotic cells was small (<40%) a finding previously reported by us, and by others, in experiments with other cell lines [13,14]. In order to ensure that the experiments were in fact measuring apoptosis, we also assessed apoptosis by TUNEL staining. The finding that following treatment with TSC, CSC and hydrogen peroxide many of the detached cells were TUNEL positive indicates that DNA damage has been induced by treatment and is likely to be associated with apoptosis since TUNEL measures nicks, or breaks, in DNA that are associated with apoptosis. However, the presence of DNA nicks by itself does not necessarily indicate apoptosis since late stage necrosis has also been associated with DNA nicks and damage [15].

In the present study, neither paraldehyde nor nicotine had any significant effect on cell death as measured by the parameters measured. These data suggest that nicotine or aldehyde, present in tobacco smoke, is unlikely to be responsible for the changes we observed following tobacco smoke exposure (see summary of treatments in table 1). In fact, the maximum concentration of nicotine used in the present study (10^-3 ^M) was 100,000 times greater than would be found physiologically (10^-8 ^M) [16] suggesting that *in vivo*, nicotine is unlikely to have an effect on lung epithelial apoptosis or cell death.

**Table 1:** A summary of apoptosis induced in A549 cells by Taxol, Tobacco smoke condensate (TSC), paraldehyde (Para), nicotine, hydrogen peroxide (HP) and fresh cigarette smoke extract (CSE) as assessed by different markers of apoptosis. Ticks () show statistically significant increases from control cells (, p < 0.05; , p < 0.01; , p < 0.001), and crosses () indicate no change from control.

	Taxol	TSC	Para	Nicotine	HP	100% CSE	50% CSE
**Cell detachment **(after 48 hrs)							
**Apoptotic morphology of detached cells **(after 48 hours)							
**TUNEL on detached cells **(after 24 hours)							
**Cytochrome C **(after 4 hours)							
**Bax-α **(after 4 hours)							

In the present study, shortly after exposure to tobacco smoke (TSC and CSE) and hydrogen peroxide, a marked release of cytochrome C from the mitochondria into the cytosol was noted. This difference was striking when measured by Western blotting but less marked when cytochrome C was measured by ELISA. This discrepancy may simply reflect the increased sensitivity of ELISA for cytochrome C as shown by the smaller amount of protein used when cytochrome C was measured by ELISA. Loss of cytochrome C from the mitochondria suggests that tobacco smoke initiates apoptosis through the mitochondrial pathway [17]. These data also suggest that, in addition to molecules such as free radicals, and aldehydes, tobacco smoke condensate may contain relatively stable molecules that also provoke cytochrome C release from mitochondria and initiate apoptosis. Furthermore, that this occurs 4 h after exposure suggests that, in our experiments, any detachment is a consequence of apoptosis initiated by release of cytochrome C, rather than apoptosis being initiated by cell detachment. However further work is necessary to determine whether or not cell detachment occurs once apoptosis is initiated or whether these two events occur in parallel.

Bax-α is a member of the Bcl-2 protein family, which are involved in controlling apoptotic events. Thus, high levels of Bcl-2 inhibit apoptosis by preventing cytochrome C release while high levels of Bax-α induce apoptosis by binding to the mitochondrial membrane and increase the permeability allowing the release of cytochrome C [17]. The present study demonstrated that cytochrome C was released from mitochondria 4 hours after exposure to CSC and TSC. Bax-α activation has been shown to occur almost simultaneously with cytochrome C loss [18] and was, therefore, assessed at 4 hours also. Bax-α was increased in the mitochondrial fraction of the cells after exposure to hydrogen peroxide and 100% CSE although there was little change in cytosolic levels. However, the total amount of Bax-α was greater in the cytosol than in the mitochondria. If the increase in Bax-α measured in the present study were to be expressed as a percentage of the total amount of Bax-α present then the change is small. It is possible that this change results in detachment and apoptosis of a relatively small percentage of cells. However, further work would be necessary to investigate this hypothesis.

The findings of the present study suggest that following exposure to tobacco smoke, Bax-α is activated prompting cytochrome C release from the mitochondria and that this may lead to DNA damage, apoptosis and cell detachment. However, pathways must exist to modulate this process since, in the present study, and that reported by others [13], substantial release of cytochrome C resulted in a comparatively modest degree of apoptosis as measured morphologically. Such pathways may also be responsible for the repair to damaged DNA seen in experiments where removal of exposure to cigarette smoke resulted in recovery of cells from damage [7].

In summary, the present study indicates that apoptosis can occur in A549 cells following exposure to tobacco smoke. Following treatment with tobacco smoke and hydrogen peroxide expression of early apoptotic markers was seen (Bax-α, and cytochrome C) 4 hours after exposure. However, when the morphology was analysed 48 hours after exposure there was a much lesser degree of apoptosis was detectable than would be suggested by the level of early marker expression. This may suggest that there are tight control mechanisms that prevent completion of the apoptotic pathway for many cells or that as the pathway progresses the cells convert to a necrotic type of cell death.

In all the experiments carried out the effect of TSC and CSE were similar except for the effect on cytochrome C release. This suggests that cell detachment, changes in morphology and DNA damage occur as a result of non-volatile components within the cigarette smoke. Whereas high levels of cytochrome C release may be due to volatile components present in the freshly prepared CSE, such as free radicals, which are unlikely to be present in the TSC.

The data shown in the present study concur with physiological characteristics of smoking related diseases. Our work suggests that although the majority of cells detaching are not necessarily apoptotic, many display apoptotic markers, in agreement with previous studies. The experiments described were conducted in a cell line which is "immortal" and may have defects in cell machinery. However, freshly isolated epithelial cells may be more sensitive than immortal cell lines to exposure to cigarette smoke and, therefore, it is possible that a larger apoptotic effect would be observed. The number of cells detaching could result in impairment of normal epithelial function, with reduced surfactant production, reduced physical defence and increased permeability of the alveolar space and inflammation all of which are common characteristics of COPD particularly emphysema. Similar findings to those described above have been reported in a recent study of lung tissue from patients with pulmonary emphysema [8] which indicates that our results are representative of events that can occur as a result of inhalation of cigarette smoke. Therefore, we conclude that tobacco smoke initiates apoptosis in airway epithelial cells as a result of mitochondrial damage and that this results in a cell detachment and full apoptosis. However, further work is necessary to define the pathways that modulate this process.

## Supplementary Material

Additional File 1 Cell morphology of detached A549 cells after 48 hour treatment. (A) control, (B) Taxol (1 μM), (C) TSC (80 μg/ml), (D) Hydrogen peroxide, (E) 50% CSE and (F) 100% CSE. Arrows indicate (1) Normal cells, (2) Indeterminate cells treated with Taxol, (3) Apoptotic cells, (4) Indeterminate cells not treated with Taxol. Click here for file
