# Supplementary figures and images for: Induction of apoptosis with tobacco smoke and related products in A549 lung epithelial cells in vitro
Source: J Inflamm (Lond). 2006 Mar 21;3:3. doi: 10.1186/1476-9255-3-3 (PMC1462990; doi:10.1186/1476-9255-3-3)

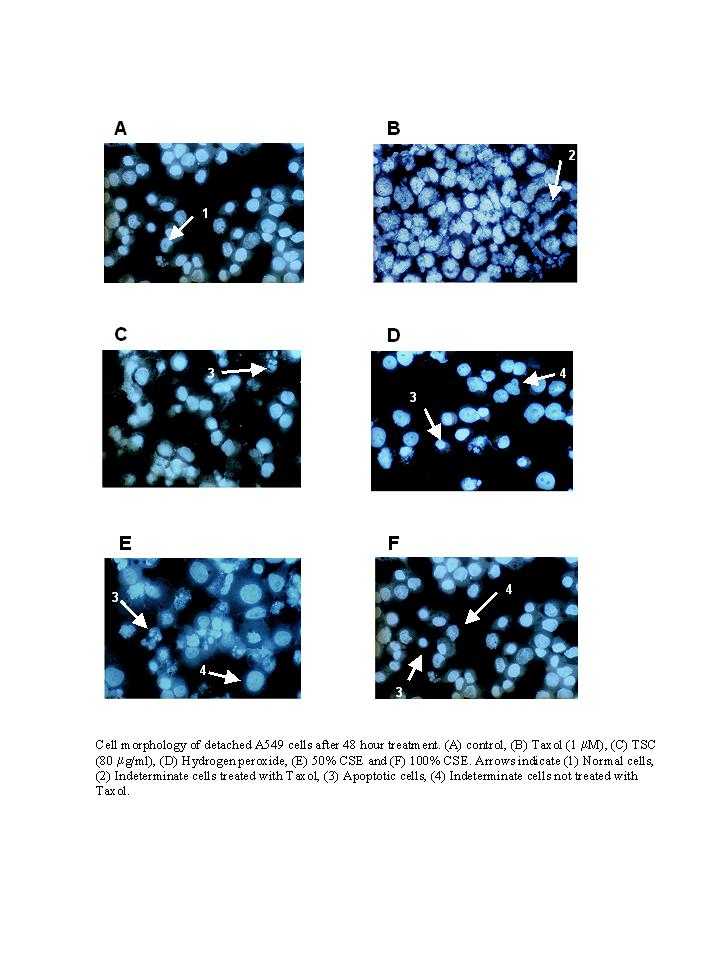

Supplement: Additional File 1 — Cell morphology of detached A549 cells after 48 hour treatment. (A) control, (B) Taxol (1 μM), (C) TSC (80 μg/ml), (D) Hydrogen peroxide, (E) 50% CSE and (F) 100% CSE. Arrows indicate (1) Normal cells, (2) Indeterminate cells treated with Taxol, (3) Apoptotic cells, (4) Indeterminate cells not treated with Taxol. [file 1476-9255-3-3-S1.JPEG]
